# Proximity to Natural Gas Wells and Reported Health Status: Results of a Household Survey in Washington County, Pennsylvania

**DOI:** 10.1289/ehp.1307732

**Published:** 2014-09-10

**Authors:** Peter M. Rabinowitz, Ilya B. Slizovskiy, Vanessa Lamers, Sally J. Trufan, Theodore R. Holford, James D. Dziura, Peter N. Peduzzi, Michael J. Kane, John S. Reif, Theresa R. Weiss, Meredith H. Stowe

**Affiliations:** 1 Yale University School of Medicine, Yale University, New Haven, Connecticut, USA; 2 University of Washington, Seattle, Washington, USA; 3 Yale School of Public Health, and; 4 Yale School of Forestry & Environmental Sciences, Yale University, New Haven, Connecticut, USA; 5 Colorado State University College of Veterinary Medicine & Biomedical Sciences, Colorado State University, Fort Collins, Colorado, USA

## Abstract

Background: Little is known about the environmental and public health impact of unconventional natural gas extraction activities, including hydraulic fracturing, that occur near residential areas.

Objectives: Our aim was to assess the relationship between household proximity to natural gas wells and reported health symptoms.

Methods: We conducted a hypothesis-generating health symptom survey of 492 persons in 180 randomly selected households with ground-fed wells in an area of active natural gas drilling. Gas well proximity for each household was compared with the prevalence and frequency of reported dermal, respiratory, gastrointestinal, cardiovascular, and neurological symptoms.

Results: The number of reported health symptoms per person was higher among residents living < 1 km (mean ± SD, 3.27 ± 3.72) compared with > 2 km from the nearest gas well (mean ± SD, 1.60 ± 2.14; *p* = 0.0002). In a model that adjusted for age, sex, household education, smoking, awareness of environmental risk, work type, and animals in house, reported skin conditions were more common in households < 1 km compared with > 2 km from the nearest gas well (odds ratio = 4.1; 95% CI: 1.4, 12.3; *p* = 0.01). Upper respiratory symptoms were also more frequently reported in persons living in households < 1 km from gas wells (39%) compared with households 1–2 km or > 2 km from the nearest well (31 and 18%, respectively) (*p* = 0.004). No equivalent correlation was found between well proximity and other reported groups of respiratory, neurological, cardiovascular, or gastrointestinal conditions.

Conclusion: Although these results should be viewed as hypothesis generating, and the population studied was limited to households with a ground-fed water supply, proximity of natural gas wells may be associated with the prevalence of health symptoms including dermal and respiratory conditions in residents living near natural gas extraction activities. Further study of these associations, including the role of specific air and water exposures, is warranted.

Citation: Rabinowitz PM, Slizovskiy IB, Lamers V, Trufan SJ, Holford TR, Dziura JD, Peduzzi PN, Kane MJ, Reif JS, Weiss TR, Stowe MH. 2015. Proximity to natural gas wells and reported health status: results of a household survey in Washington County, Pennsylvania. Environ Health Perspect 123:21–26; http://dx.doi.org/10.1289/ehp.1307732

## Introduction

Unconventional methods of natural gas extraction, including directional drilling and hydraulic fracturing (also known as “fracking”), have made it possible to reach natural gas reserves in shale deposits thousands of feet underground (Myers 2012). Increased drilling activity in a number of locations in the United States has led to growing concern that natural gas extraction activities could contaminate water supplies and ambient air, resulting in unforeseen adverse public health effects (Goldstein et al. 2012). At the same time, there is little peer-reviewed evidence regarding the public health risks of natural gas drilling activities (Kovats et al. 2014; McDermott-Levy and Kaktins 2012; Mitka 2012), including a lack of systematic surveys of human health effects.

*The process of natural gas extraction*. Natural gas extraction of shale gas reserves may involve multiple activities occurring over a period of months. These include drilling and casing of deep wells that contain both vertical and horizontal components as well as placement of underground explosives and transport and injection of millions of gallons of water containing sand and a number of chemical additives into the wells at high pressures to extract gas from the shale deposits (hydraulic fracturing) (Jackson RE et al. 2013). Chemicals used in the hydraulic fracturing process can include inorganic acids, polymers, petroleum distillates, anti-scaling compounds, microbicides, and surfactants (Vidic et al. 2013). Although some of these fluids are recovered during the fracking process as “flowback” or “produced” water, a significant amount (as much as 90%) (Vidic et al. 2013) may remain underground. The recovered flowback water—which may contain chemicals added to the fracking fluid as well as naturally occurring chemicals such as salts, arsenic, and barium and naturally occurring radioactive material originating in the geological formations—may be stored in holding ponds or transported offsite for disposal and/or wastewater treatment elsewhere.

*Potential water exposures*. Although much of the hydraulic fracturing process takes place deep underground, there are a number of potential mechanisms for chemicals used in the fracturing process as well as naturally occurring minerals, petroleum compounds (including volatile organic compounds; VOCs), and other substances of flowback water (Chapman et al. 2012) to enter drinking-water supplies. These include spills during transport of chemicals and flowback water, leaks of a well casing (Kovats et al. 2014), leaks through underground fissures in rock formations, runoff from drilling sites, and disposal of fracking flowback water (Rozell and Reaven 2012). Studies have reported increased levels of methane in drinking water wells located < 1 km from natural gas drilling, suggesting contamination of water wells from hydraulic fracturing activities (Jackson RB et al. 2013; Osborn et al. 2011), although natural movement of methane and brine from shale deposits into aquifers has also been suggested (Warner et al. 2012). If contaminants from hydraulic fracturing activities were able to enter drinking water supplies or surface water bodies, humans could be exposed to such contaminants through drinking, cooking, showering, and swimming.

*Potential air exposures*. The drilling and completion of natural gas wells, as well as the storage of waste fluids in containment ponds, may release chemicals into the atmosphere through evaporation and off-gassing. In Pennsylvania, flowback fluids are not usually disposed of in deep injection wells; therefore surface ponds containing flowback fluids are relatively common and could be sources of air contamination through evaporation. Flaring of gas wells, operation of diesel equipment and vehicles, and other point sources for air quality contamination around drilling activities may also pose a risk of respiratory exposures to nitrogen oxides, VOCs, and particulate matter. Release of ozone precursors into the environment by natural gas production activities may lead to increases in local ozone levels (Olaguer 2012). Well completion and gas transport may cause leakage of methane and other greenhouse gases into the environment (Allen 2014). Studies in Colorado have reported elevated air levels of VOCs including trimethylbenzenes, xylenes, and aliphatic hydrocarbons related to well drilling activities (McKenzie et al. 2012).

*Human health impact*. Concerns about the impact of natural gas extraction on the health of nearby communities have included exposures to contaminants in water and air described above as well as noise and social disruption (Witter et al. 2013). A published case series cited the occurrence of respiratory, skin, neurological, and gastrointestinal symptoms in humans living near gas wells (Bamberger and Oswald 2012). A convenience sample survey of 108 individuals in 55 households across 14 counties in Pennsylvania who were concerned about health effects from natural gas facilities found that a number of self-reported symptoms were more common in individuals living near gas facilities, including throat and nasal irritation, eye burning, sinus problems, headaches, skin problems, loss of smell, cough, nosebleeds, and painful joints (Steinzor et al. 2013). Similarly, a convenience sample survey of 53 community members living near Marcellus Shale development found that respondents attributed a number of health impacts and stressors to the development. Stress was the symptom reported most frequently (Ferrar et al. 2013).

Here we report on the analysis of a cross-sectional, random-sample survey of the health of residents who had ground-fed water wells in the vicinity of natural gas extraction wells to determine whether proximity to gas wells was associated with reported respiratory, dermal, neurological, or gastrointestinal symptoms.

## Methods

**Figure 1:**
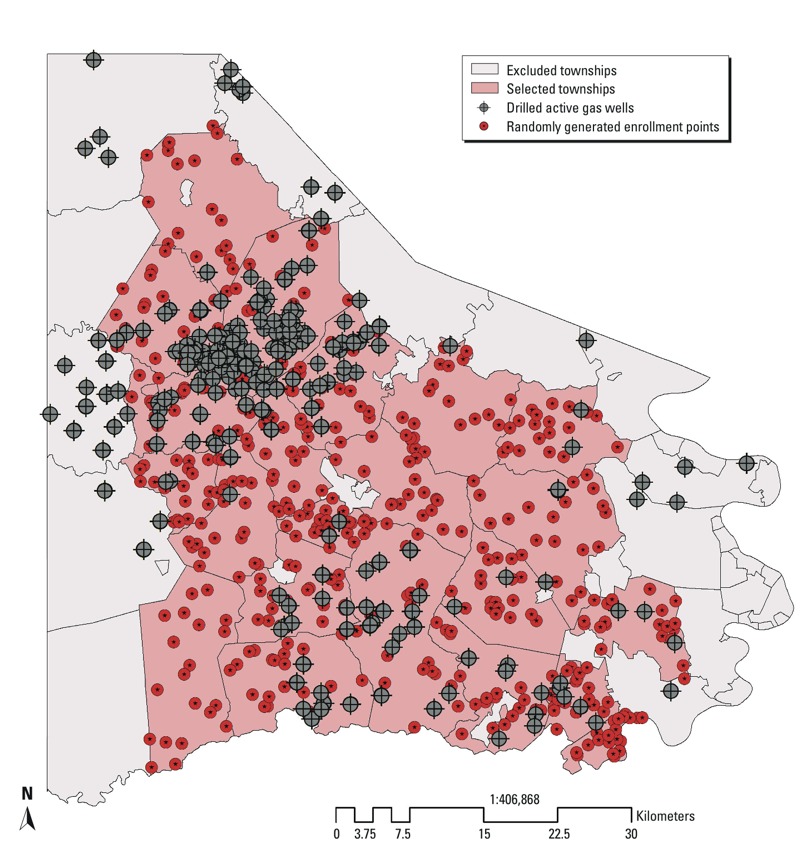
Distribution of drilled active Marcellus Shale natural gas wells (*n* = 624) and randomly generated sampling sites (*n* = 760) for eligible municipalities of Washington County, Pennsylvania.

*Selection of study area*. The Marcellus formation, a principal source of shale-based natural gas in the United States, is a Middle Devonian–age black, low-density, organically rich shale that has been predominantly horizontally drilled for gas extraction in the southwestern portion of Pennsylvania since 2003 [Pennsylvania Spatial Data Access (PASDA) 2013]. In this study we focused on Washington County in southwestern Pennsylvania, an area of active natural gas drilling (Carter et al. 2011). At the time of the administration of the household survey during summer 2012, there were, according to the Pennsylvania Department of Environmental Protection, 624 active natural gas wells in Washington County. Of these natural gas wells, 95% were horizontally drilled (Pennsylvania Department of Environmental Protection 2012). The county has a highly rural classification with nearly 40% of the land devoted to agriculture (U.S. Department of Agriculture 2007). Washington County has a population of approximately 200,000 persons with 94% self-identified as white, 90% having at least a high school diploma, and a 2012 median household income of $53,545 (Center for Rural Pennsylvania 2014). We selected a contiguous set of 38 rural townships within the center of Washington County as our study site in order to avoid urban areas bordering Pittsburgh, which would be unlikely to have ground-fed water wells, and areas near the Pennsylvania border, which might be influenced by gas wells in other states (Figure 1).

*Survey instrument*. We designed a community environmental health assessment of reported health symptoms and health status based on questions drawn from publicly available surveys. Symptom questions, covering a range of organ systems that had been mentioned in published reports (Bamberger and Oswald 2012; Steinzor et al. 2013), asked respondents whether they or any household members had experienced each condition during the past year (see Supplemental Material, “Questionnaire”). The health assessment also asked a number of general yes/no questions about concerns of environmental hazards in the community, such as whether respondents were satisfied with air quality, water quality, soil quality, environmental noise and odors, and traffic, but did not specifically mention natural gas wells or hydraulic fracturing or other natural gas extraction activities. The survey was pretested with focus groups in the study area in collaboration with a community based group and revised to ensure comprehensibility of questions.

*Selection and recruitment of households*. Using ArcGIS Desktop 10.0 software (ESRI, Inc., Redlands, CA), we randomly selected 20 geographic points from each of 38 contiguous townships in the study county (Figure 1). We identified an eligible home nearest to each randomly generated sampling point, and visited each home to determine which households were occupied and had ground-fed water wells. We selected households with ground-fed water wells to assess possible health effects related to water contamination. From the original 760 points identified (i.e., 20 points in each of the 38 townships), we excluded 12 duplicate points and 64 points found not to correspond to a house structure (see Supplemental Material, Figure S1). After site visits by the study team who spoke to residents or neighbors, we excluded house locations determined not to have a ground-fed well or spring. Additional points were excluded if the structure was not occupied (*n* = 5) or inaccessible from the road (*n* = 4). During visits to eligible households, a study member invited a responding adult at least 18 years of age to participate in the survey, described as a survey of community environmental health that considered a number of environmental health factors. Three households were excluded when the respondent was unable to answer the questionnaire due to language or health problems. Eligible households were offered a small cash stipend for participation.

The Yale University School of Medicine Human Research Protection Program determined the study to be exempt from Human Subjects review. Respondents provided oral consent but were not asked to sign consent forms; their names were not recorded.

Of the 255 eligible households, respondents refused to complete the survey in 47 households, and we were not able to contact residents in another 26 households. Reasons for refusal included “not interested” (*n* = 8), “no time/too busy” (*n* = 3), “afraid” (*n* = 1), and 35 gave no reason. The rate of refusal varied by distance category, with 12 of 74 (16%) of households < 1 km from a gas well, 10 of 67 (15%) of households 1–2 km from wells, and 25 of 86 (25%) of eligible households > 2 km from a gas well refusing to participate, but the differences were not statistically significant. At the consenting 180 households (71% of eligible households), an adult respondent completed the survey covering the health status of the 492 individuals living in these households.

*Administration of survey at residence*. Trained study personnel administered the survey in English. The responding adult at the participating household reported on the health status of all persons in the household over the past year. A study team member recorded the global positioning system (GPS) coordinates of the household using a Garmin GPSMAP® 62S Series handheld GPS device (Garmin International, Inc., Olathe, KS). Survey personnel were not aware of the mapping results for gas well proximity to the households being surveyed.

*Household proximity to nearest active gas well and age of wells*. A map of 624 active natural gas wells in the study area, and their age and type, was created by utilizing gas well permit data publicly available at the PASDA (2013). Ninety five percent of the gas wells had “spud dates” (first date of drilling) between 2008 and 2012, with more than half of spud dates occurring in 2010 and 2011. We used ArcGIS to calculate the distance between each household location (as defined by the GPS reading taken during the site visit) and each natural gas well in the study area. We then classified households according to their distance from the nearest gas well with distance categories of < 1 km, 1–2 km, or > 2 km. We used 1 km as the initial cut point for distance to a nearest gas well because of the reported association of higher methane levels in drinking-water wells located < 1 km from natural gas wells (Osborn et al. 2011), and 2 km as the second cut point because it was close to the mean of the distances between households and nearest gas wells. The mean and median distance between a household and the nearest natural gas well were 2.0 km and 1.4 km, respectively. We classified the age of each gas well as the time interval between spud date and the date that the household survey was conducted during summer, 2012.

*Statistical analysis*. Demographic variables were analyzed for differences among individuals between distance categories using chi-square, analysis of variance, or generalized linear mixed-model statistics as appropriate. Reported occupation was classified as either blue collar, office sales and service, management/professional, or not working, using classifications of the U.S. Bureau of Labor Statistics (2014).

The prevalence of each outcome and the number of symptoms reported for each household member included in the study were calculated according to the distance of each household (< 1, 1–2, or > 2 km) from the nearest gas well. To test the association between household distance from a well and the overall number of symptoms as well as the presence or absence of each of six groups of health conditions (dermal, upper respiratory, lower respiratory, gastrointestinal, neurological, and cardiovascular), we used SAS 9.3 in a generalized linear mixed model (GLMM) analysis (SAS Institute Inc., Cary, NC). The analysis used maximum likelihood estimation with adaptive quadrature methods (Schabenberger 2007) including a random effect for household to account for the clustering of individuals within a household. The model was adjusted for age of individual (continuous), sex (binary), average adult household education (continuous), smoker present in household (yes/no), awareness of environmental hazard nearby (yes/no), employment type (four categories), and whether animals were present in the home or backyard (yes/no). Given the exploratory nature of this study, no adjustments were made for multiple comparisons and significance was established at the two-sided 0.05 level. Statistical analyses were conducted using SAS 9.3.

## Results

*Demographics*. Individuals living in households < 1 km from gas wells were older (mean, 46.9 ± 21.9) compared with individuals in households > 2 km from a gas well (mean, 40.0 ± 23.5 years, *p* = 0.03) (Table 1). There was a higher proportion of children in the households > 2 km from a gas well compared with those < 1 km from a gas well (27% vs. 14%, *p* = 0.008). Families had lived in their homes an average of 22.8 ± 17.2 years at the time of the interview. Thirty-four percent of individuals had blue-collar jobs and 38% of the subjects were nonworkers (e.g., unemployed, students). Sixty-six percent reported using their ground-fed water (well or natural spring) for drinking water, and 84% reported using it for other activities such as bathing. The age of the nearest gas well was significantly greater for households < 1 km from a gas well (mean, 2.3 ± 1.6) compared with those 1–2 km or > 2 km from a well (1.5 ± 1.3 and 1.1 ± 0.9, respectively, *p* < 0.05). Reported smoking was less common in households near gas wells, whereas reported respondent awareness regarding environmental health risks was higher, although these differences were not statistically significant.

**Table 1:** Demographics and household characteristics by proximity to the nearest natural gas well.

Characteristic	< 1 km	1–2 km	> 2 km	All
Individuals
*n*	150	150	192	492
Sex
Male	80 (53)	78 (52)	92 (48)	250 (51)
Female	70 (47)	72 (48)	100 (52)	242 (49)
Children	21 (14)*	27 (18)	52 (27)	100 (20)
Education (years)	13.4 ± 2.0	13.5 ± 1.9	13.3 ± 2.0	13.4 ± 1.9
Age (years)	46.9 ± 21.9**	45.5 ± 22.7	40.0 ± 23.5	43.8 ± 23.0
Occupation^*a*^
M/P	29 (19)	34 (23)	33 (17)	96 (19)
O/S	17 (11)	11 (7)	14 (7)	42 (9)
BC	60 (40)	51 (34)	56 (29)	167 (34)
NW	44 (29)	54 (36)	89 (46)	187 (38)
Households
*n*	62	57	61	180
Smoking^*b*^	7 (11)	12 (21)	14 (23)	33 (18)
Years in household (*n*)	23.7 ± 16.6	23.5 ± 16.4	21.2 ± 18.6	22.8 ± 17.2
Body mass index (kg/m^2^)	27.9 ± 5.1	27.5 ± 5.4	27.9 ± 6.1	27.8 ± 5.5
Use ground-fed water
Drinking	39 (63)	41 (72)	38 (62)	118 (66)
Other	54 (87)	51 (89)	46 (75)	151 (84)
Water has unnatural appearance	13 (21)	7 (12)	6 (10)	26 (14)
Taste/odor prevents water use	14 (23)	10 (18)	19 (31)	43 (24)
Dissatisfied with odor in environment	7 (11)	1 (2)	1 (2)	9 (5)
Environmental risk awareness^*c*^	16 (25)	16 (28)	9 (15)	41 (23)
Years since spud date of closest well (years)	2.3 ± 1.6^*#*^	1.5 ± 1.3	1.1 ± 0.9	1.6 ± 1.4
Values are *n* (%) or mean ± SD. ^***a***^Participant occupation was categorized into six main industries according to the U.S. Bureau of Labor Statistics (2014), and presented here in four main groups: M/P, management or professional; O/S, office, sales, or service; BC, blue collar (fishing, farming, and forestry; construction, extraction, maintenance, production, transportation, and material moving); NW, nonworker (student, disabled, retired, or unemployed). ^***b***^Household smoking was determined when respondents were asked if they or at least one member of their household smoked cigarettes in the house at the time of the survey. ^***c***^Household respondents were asked if they were aware of any environmental health risks near their residence (yes/no), to approximate potential sources of expectation or awareness bias. *p = 0.008 compared with > 2 km households. **p = 0.03 compared with > 2 km households. ^#^*p *< 0.05 compared with 1–2 km and > 2 km households.				

*Reported health symptoms*. The average number of reported symptoms per person in residents of households < 1 km from a gas well (3.27 ± 3.72) was greater compared with those living > 2 km from gas wells (1.60 ± 2.14, *p* = 0.0002).

Individuals living in households < 1 km from natural gas wells were more likely to report having any of the queried skin conditions over the past year (13%) than residents of households > 2 km from a well (3%; χ^2^ = 13.8, *p* = 0.001) (Table 2). Reported upper respiratory symptoms were also more frequent among households < 1 km (39%) compared with households > 2 km from gas wells (18%; χ^2^ = 17.9, *p* = 0.0001).

**Table 2:** Prevalence of selected health conditions reported by individuals by proximity to the nearest gas well (2011–2012).^*a*^

Symptoms	< 1 km (*n* = 150)	1–2 km (*n* = 150)	> 2 km (*n* = 192)
Total number of symptoms per individual	3.27 ± 3.72	2.56 ± 3.26	1.60 ± 2.14
Dermal [*n* (%)]	19 (13)	7 (5)	6 (3)
Rashes/skin problems	10 (7)	7 (5)	6 (3)
Dermatitis	6 (4)	5 (3)	2 (1)
Irritation	6 (4)	2 (1)	1 (1)
Burning	8 (5)	4 (3)	1 (1)
Itching	9 (6)	5 (3)	2 (1)
Hair loss	2 (1)	0 (0)	1 (1)
Upper respiratory [*n* (%)]	58 (39)	46 (31)	35 (18)
Allergies/sinus problems	35 (23)	27 (18)	27 (14)
Cough/sore throat	10 (7)	3 (2)	2 (1)
Itchy eyes	19 (13)	22 (15)	10 (5)
Nose bleeds	13 (9)	8 (5)	4 (2)
Stuffy nose	16 (11)	8 (5)	4 (2)
Lower respiratory [*n* (%)]	29 (19)	29 (19)	27 (14)
Asthma/COPD	16 (11)	21 (14)	15 (8)
Chronic bronchitis	8 (5)	2 (1)	2 (1)
Chest wheeze/whistling	6 (4)	9 (6)	7 (4)
Shortness of breath	8 (5)	7 (5)	8 (4)
Chest tightness	4 (3)	6 (4)	5 (3)
Cardiac [*n* (%)]	46 (31)	39 (26)	37 (19)
High blood pressure	38 (25)	33 (22)	29 (15)
Chest pain	8 (5)	5 (3)	6 (3)
Heart palpitations	10 (7)	7 (5)	4 (2)
Ankle swelling	11 (7)	5 (3)	5 (3)
Gastrointestinal [*n* (%)]	15 (10)	13 (9)	11 (6)
Ulcers/stomach problems	11 (7)	7 (5)	8 (4)
Liver problems	4 (3)	0 (0)	1 (0.5)
Nausea/vomiting	1 (1)	3 (2)	1 (0.5)
Abdominal pain	4 (3)	2 (1)	2 (1)
Diarrhea	5 (3)	2 (1)	2 (1)
Bleeding	4 (3)	4 (3)	0 (0)
Neurologic [*n* (%)]
Neurologic problems	1 (0.7)	5 (3)	0 (0)
Severe headache/migraine	24 (16)	14 (9)	18 (9)
Dizziness/balance problems	11 (7)	12 (8)	11 (6)
Depression	4 (3)	3 (2)	2 (1)
Difficulty concentrating/remembering	9 (6)	9 (6)	6 (3)
Difficulty sleeping/insomnia	18 (12)	19 (13)	10 (5)
Anxiety/nervousness	11 (7)	4 (3)	11 (6)
Seizures	2 (1)	2 (1)	1 (0.5)
COPD, chronic obstructive pulmonary disease. ^***a***^Six categories representing major health conditions of *a priori* interest chosen to ascertain symptom prevalence among individuals living in proximity to the nearest gas well in 2011–2012.			

In a hierarchical model that adjusted for age, sex, household education level, smokers in household, job type, animals in household, and awareness of environmental risk (Table 3), household proximity to natural gas wells remained associated with number of symptoms reported per person < 1 km (*p* = 0.002) and 1–2 km (*p* = 0.05) compared with > 2 km from gas wells, respectively. In similar models, living in a household < 1 km from the nearest gas well remained associated with increased reporting of skin conditions [odds ratio (OR) = 4.13; 95% confidence interval (CI): 1.38, 12.3] and upper respiratory symptoms (OR = 3.10; 95% CI: 1.45, 6.65) compared with households > 2 km from the nearest gas well.

**Table 3:** Associations of nearest gas well proximity and symptoms.

Outcome	< 1 km		1–2 km		> 2 km
	OR (95% CI)	*p*-Value	OR (95% CI)	*p*-Value	
Dermal	4.13 (1.38, 12.3)	0.011	1.44 (0.42, 4.9)	0.563	Ref
Upper respiratory	3.10 (1.45, 6.65)	0.004	1.76 (0.81, 3.76)	0.148	Ref
Lower respiratory	1.45 (0.67, 3.14)	0.339	1.40 (0.65, 3.03)	0.387	Ref
Cardiac	1.67 (0.85, 3.26)	0.135	1.28 (0.65, 2.52)	0.473	Ref
Gastrointestinal	2.01 (0.49, 8.18)	0.328	1.79 (0.43, 7.41)	0.417	Ref
Neurological	1.53 (0.89, 2.63)	0.123	1.04 (0.59, 1.82)	0.885	Ref
Ref, reference.^******^Results are from hierarchical logistic regression that adjusted for age, household education level, sex, smokers in household, job type, animals in household, and awareness of environmental risk.					

For the other grouped symptom complexes examined, there was not a significant relationship in our adjusted model between the prevalence of symptom reports and proximity to nearest gas well. In the multivariate model, however, environmental risk awareness was significantly associated with report of all groups of symptoms.

Age of the nearest gas well was found to be negatively correlated with distance (*r* = –0.325; *p* < 0.0001): Gas wells < 1 km from households tended to be older than the nearest wells in other distance categories. When age of wells was added to the multivariate model, proximity to gas wells remained significantly associated with respiratory symptoms, but the association between proximity and dermal symptoms lost statistical significance.

## Discussion

This spatially random health survey of households with ground-fed water supply in a region with a large number of active natural gas wells is to our knowledge the largest study to date of the association of reported symptoms and natural gas drilling activities.We found an increased frequency of reported symptoms over the past year in households in closer proximity to active gas wells compared with households farther from gas wells. This association was also seen for certain categories of symptoms, including skin conditions and upper respiratory symptoms. This association persisted even after adjusting for age, sex, smokers in household, presence of animals in the household, education level, work type, and awareness of environmental risks. Other groups of reported symptoms, including cardiac, neurological, or gastrointestinal symptoms, did not show a similar association with gas well proximity. These results support the need for further investigation of whether natural gas extraction activities are associated with community health impacts.

These findings are consistent with earlier reports of respiratory and dermal conditions in persons living near natural gas wells (Bamberger and Oswald 2012; Steinzor et al. 2013). Strengths of the study included the larger sample size compared with previously published surveys, and the random method of selecting households using geographic information system methodology, which reduces the possibility of selection bias (although only a subset of households, those with ground-fed water supply, were sampled).

A limitation of the study was the reliance on self-report of health symptoms. On one hand, symptoms in other household members may have been underreported by the household respondent; on the other hand, awareness bias in individuals concerned about the presence of an environmental health hazard would be more likely to increase reporting of illness symptoms, leading to recall bias of the results. We did not collect data on whether individuals were receiving financial compensation for gas well drilling on their property, which could have affected their willingness to report symptoms. It is possible that differential refusal to participate could have introduced potential for selection bias; for example, individuals who were receiving compensation for gas drilling on their property might be less willing to participate in the survey. We found instead that the refusal rate, though < 25% overall, was higher among households farther from gas wells, suggesting that such households may have been less interested in participating because they had less awareness of hazards. The study questionnaire did not include questions about natural gas extraction activities, in order to reduce awareness bias. At the same time, it is likely that household residents were aware of gas drilling activities in the vicinity of households; and the fact that reported environmental awareness by respondents was associated with the prevalence of all groups of reported health symptoms suggests a correlation between heightened awareness of health risks and reported health conditions. Nevertheless, the observed association between gas well proximity and reported dermal and upper respiratory symptoms persisted in the multivariate model even after adjusting for environmental awareness. Future studies should attempt to medically confirm particular diagnoses and further assess and control for the effect of awareness on reported health status.

A further study limitation was the fact that our analysis includes multiple comparisons between groups of households, and the consequent possibility that random error could account for some of our findings. We limited such comparisons by grouping individual symptoms into organ system clusters. However, we acknowledge that the multiple comparisons used in the methodology mean that any such particular findings should be viewed as preliminary and hypothesis generating.

Our use of gas well proximity as a measure of exposure was an indirect measure of potential water or airborne exposures. More precise data could come from direct monitoring and modeling of air and water contaminants, and correlating such measured exposures with confirmed health effects should be a focus of future study. Biomonitoring of individuals living near natural gas wells could provide additional information about the role and extent of particular chemical exposures.

There are several potential explanations for the finding of increased skin conditions among inhabitants living near gas wells. One is that natural gas extraction wells could have caused contamination of well water through breaks in the gas well casing or other underground communication between ground water supplies and fracking activities. The geographic area studied has experienced petroleum and coal exploration and extraction activities in the past century, and such activities may increase the risk of chemicals in fracking fluid or flowback water entering ground water and contaminating wells. If such contamination did occur, several types of chemicals in fracking fluid have irritant properties and could potentially cause skin rashes or burning sensation through exposure during showers or baths. There are published reports of associations between the prevalence of eczema and other skin conditions with exposure to drinking water polluted with chemicals including VOCs (Chaumont et al. 2012; Lampi et al. 2000; Yorifuji et al. 2012) as well as changes in water hardness (Chaumont et al. 2012; McNally et al. 1998).

A second possible explanation for the skin symptoms could be exposure to air pollutants including VOCs, particulates, and ozone from upwind sources, such as flaring of gas wells (McKenzie et al. 2012) and exhaust from vehicles and heavy machinery.

A third possibility to explain the clustering of skin and other symptoms would be that they could be related to stress or anxiety that was greater for households living near gas wells. In this study, awareness of environmental risk was independently associated with overall reporting of symptoms as well as reporting of skin problems. However, in multivariate models, proximity to gas wells remained a significant predictor of symptoms even when adjusting for such awareness. These results argue for possible air or water contaminant exposures, in addition to stress, contributing to the observed patterns of increased health symptoms in households near gas wells. A fourth possibility would be the role of allergens or irritant chemicals not related to natural gas drilling activities, such as exposure to agricultural chemicals or household animals. We did not see a correlation between skin conditions and either the presence of an animal in the household or agricultural occupation, making this association less likely. At the same time, it is possible that other confounding could be present but not accounted for in our models.

Our findings of increased reporting of upper respiratory symptoms among persons living < 1 km from a natural gas well suggests that airborne irritant exposures related to natural gas extraction activities could be playing a role. Such irritant exposures could result from a number of activities related to natural gas drilling, including flaring of gas wells and exhaust from diesel equipment. Because other studies have suggested that airborne exposures could be a significant consequence of natural gas drilling activity, further investigation of the impact of such activities on respiratory health of nearby communities should be investigated. Future studies should collect such data.

Since most of the gas wells in the study area had been drilled in the past 5–6 years, one would not yet expect to see associations with diseases with long latency, such as cancer. Furthermore, if some of the impact of natural gas extraction on ground water happens over a number of years, this initial survey could have failed to detect health consequences of delayed contamination. However, if the finding of skin and respiratory conditions near gas wells indicates significant exposure to either fracking fluids and chemicals or airborne contaminants from natural gas wells, studies looking at such long-term health effects in chronically exposed populations would be indicated.

## Conclusions

The results of this study suggest that natural gas drilling activities could be associated with increased reports of dermal and upper respiratory symptoms in nearby communities; these results support the need for further research into health effects of natural gas extraction activities. Such research could include longitudinal assessment of the health of individuals living in proximity to natural gas drilling activities, medical confirmation of health conditions, and more precise assessment of contaminant exposures.

## Supplemental Material

(659 KB) PDF
