# Supplementary material for: Proximity to Natural Gas Wells and Reported Health Status: Results of a Household Survey in Washington County, Pennsylvania
Source: Environ Health Perspect. 2014 Sep 10;123(1):21–6. doi: 10.1289/ehp.1307732 (PMC4286272; doi:10.1289/ehp.1307732)
Supplement: (659 KB) PDF [file ehp.1307732.s001.508.pdf]

Environmental Health Perspectives doi:10.1289/ehp.1307732

Supplemental Material

Proximity to Natural Gas Wells and Reported Health Status: Results of a Household Survey in Washington County, Pennsylvania

Peter M. Rabinowitz, Ilya B. Slizovskiy, Vanessa Lamers, Sally J. Trufan, Theodore R. Holford, James D. Dziura, Peter N. Peduzzi, Michael J. Kane, John S. Reif, Theresa R. Weiss, and Meredith H. Stowe

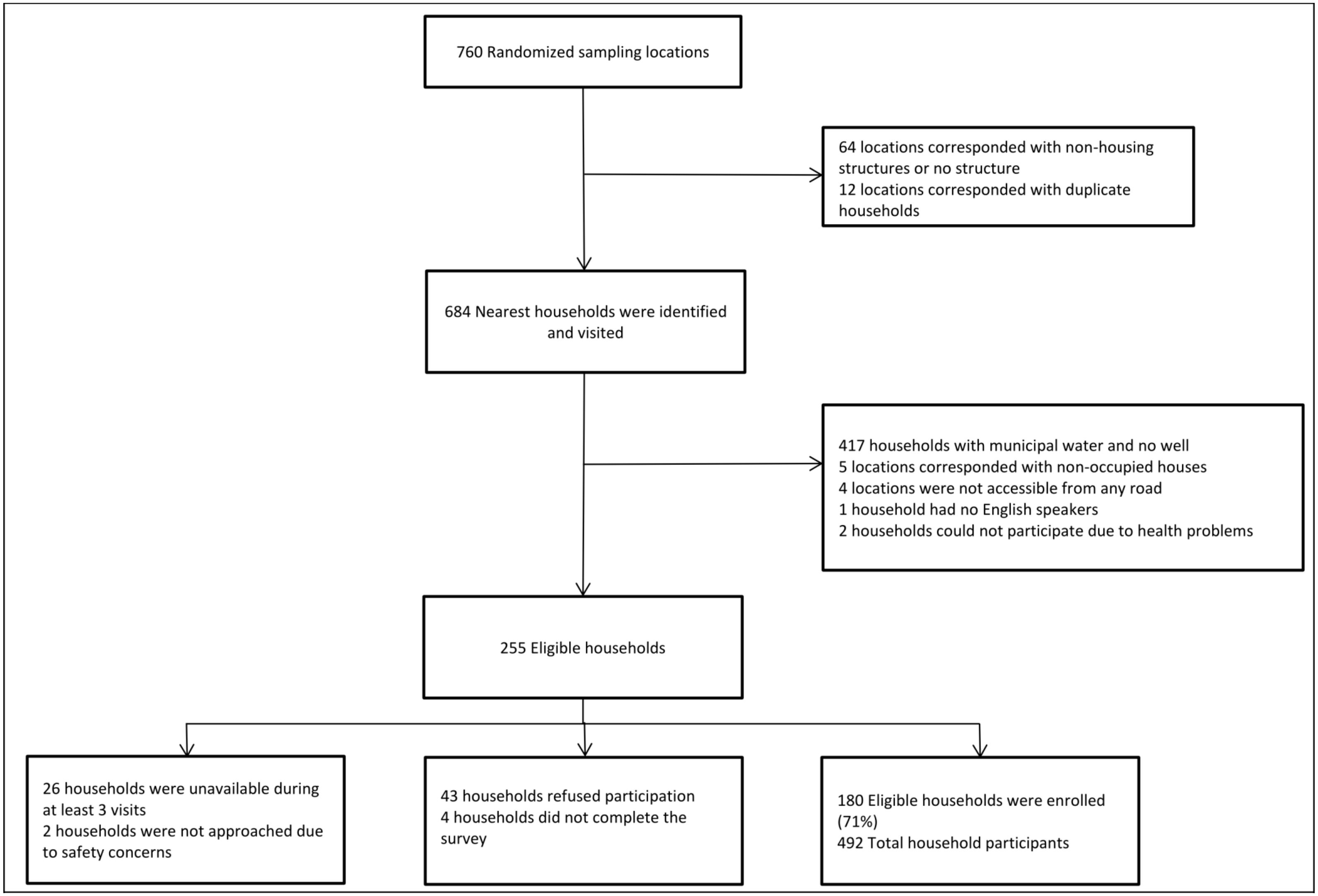

Figure S1. Screening, Enrollment, and Survey

From the original 760 random points geospatially identified, 417 house locations were determined to have municipal water supply and no well; 12 others were not eligible. Forty-seven of the remaining 255 eligible households refused to complete the survey; at 26 houses respondents were not available at multiple follow-up visits; 2 were not approached for safety reasons. At the remaining 180 households (71% of the 255 eligible households), an adult respondent agreed to complete the survey covering the health status of the 492 individuals living in these households.

Supplemental Material: Questionnaire

Examples of questions asked on the survey and included in analysis or demographic tables

Length of residence

How long have you (or anyone in the household) lived at this residence? _________ years 77 RF 99 DK

Demographics

I would like to ask about each member of your household, but please don't give me any names – just first initial.

Let's start with you.

a.Initials	b.Gender (M or F)	c.Age	d.Education (highest grade completed)	Current job: e. type of work f. job title
1				
2				
3 ….				

Smoking

Does anyone in this household smoke regularly inside the house? 1 YES 2 NO 7 RF 9 DK

Biometrics

What is your height? _______________ inches

What is your weight? _________________ pounds

Personal Health Symptoms

Have you or anyone in the household had any of the following health conditions in the past year?

CONDITION	a. Have you had? 1Yes 2No 7RF 9DK	b. INITIALS OF anyone else?
**RESPIRATORY ALLERGY/ENT:**		
1 Asthma/COPD		
2 Allergies/Sinus problems		
3 Metallic taste in the mouth		
4 Chronic bronchitis		
5 Chest Wheezing or whistling		
6 Shortness of breath		
7 Chest tightness lasting for ≥ 30 min		
8 Cough/sore throat not caused by a cold lasting for ≥ 14 days		
9 Frequent itchy or irritated eyes		
10 Frequent nose bleeds		
11 Stuffy nose/rhinitis		
12 Hearing loss or dysfunction		
13 Tinnitus/ringing in the ears		
**DERMAL:**		
14 Rashes or skin problems		
15 Dermatitis		
16 Skin irritation (over areas of the body that stayed for 3 days or greater than **does not** include measles, chicken pox, poison ivy/oak, bites, or allergic reaction)		
17 Burning skin		
18 Itching		
19 Hair loss		
**CARDIAC:**		
20 High blood pressure		
21 Chest pain		
22 Heart palpitations		
23 Ankle swelling		
**MUSCULOSKELETAL:**		
24 Muscle weakness/or pain		
25 Joint pain, aching, or stiffness		
**NEUROLOGIC:**		
26 Neurologic problems		
27 Severe headache or migraine		
28 Dizziness/balance problems		
29 Depression		
30 Difficulty concentrating/remembering		
31 Regular difficulty sleeping and/or insomnia		
32 Anxiety/nervousness		
33 Seizures		

CONDITION	a. Have you had? 1Yes 2No 7RF 9DK	b. INITIALS OF anyone else?
34 Substance abuse (other than alcohol and tobacco)?		
35 Excessive alcohol problems?		
**GI/LIVER:**		
36 Stomach problems/ulcers		
37 Any kind of liver condition		
38 Nausea/vomiting		
39 Abdominal pain		
40 Diarrhea		
41 Bleeding		
**KIDNEY:**		
42 Kidney disease or problems		
43 Weak or failing kidney(s) (excluding kidney stones, bladder infections, or incontinence)		
**REPRODUCTIVE:**		
44 Infertility		
45 Birth defects		
46 Unusual vaginal bleeding [heavy bleeding, bothersome cramping, or pre-menstrual syndrome (PMS)]		
47 Miscarriage/still birth		
**ENDOCRINE:**		
48 Weight loss or gain		
49 Thyroid problems		
**BLOOD:**		
50 Leukemia/lymphoma		
51 Anemia		
52 Swollen glands		
53 Unusual bleeding		
**OTHER:**		
54 Fatigue or lack of energy for ≥ 3 days		
55 Other health problems:_____________		

Environmental Quality

For these questions, there is no right or wrong answer.

For each of the following, please choose the answer on side 1 of the blue card that best describes how **satisfied** you are with that area of your personal life or your community/neighborhood. [Very dissatisfied, moderately dissatisfied, slightly dissatisfied, slightly satisfied, moderately satisfied, very satisfied]

1. How *satisfied* are you with:

a. Safe areas for walking/jogging/biking?	
b. Air quality/pollution?	
c. Water quality/pollution?	
d. Land or soil quality/pollution?	
e. Neighborhood level of noise?	
f. Environmental odor(s)?	
g. Neighborhood road use and traffic?	

Residential Environment

Do you have any pets? 1 YES 2 NO 7 RF 9 DK

Do you have any farm animals? 1 YES 2 NO 7 RF 9 DK

Are you aware of any environmental health risks near your residence? 1 YES 2 NO 7 RF 9 DK

Household Water

What is the **main** source of drinking-water for members of your household?

1 PUBLIC TAP
2 WELL, UNSPECIFIED
2a DRILLED WELL
2b PROTECTED DUG WELL
2c UNPROTECTED DUG WELL
3 SPRING
4 BOTTLED WATER [a. date started? _______]
5 TANK/CISTERN [a. date started?_________]
6 WATER BUFFALO [a. date started?_________]
8 OTHER: ____________________________________
7 RF
9 DK

What is the **main** source of water used by your household for other purposes, such as laundry, hand-washing, and showering?

1 PUBLIC TAP
2 WELL, UNSPECIFIED
2a DRILLED WELL
2b PROTECTED DUG WELL
2c UNPROTECTED DUG WELL
3 SPRING
4 BOTTLED WATER [a.date started? ________]
5 TANK/CISTERN [a.date started?_________]
6 WATER BUFFALO [a.date started?_________]
8 OTHER: ____________________________________
7 RF
9 DK

Does the taste or odor of your well water prevent you or members of your household from drinking, cooking, bathing (etc.), or otherwise using the water that is piped to your premises?

1 YES 2 NO 3 SOMETIMES 7 RF 9 DK

Does your well water have an unnatural color or appearance?

1 YES 2 NO 3 SOMETIMES 7 RF 9 DK

Which racial or ethnic group do you most identify with?

1 American Indian or Alaskan Native

2 Asian

3 Black or African American

4 Native Hawaiian or Pacific Islander

5 Hispanic/Latino

6 Caucasian/European descent/White

8 Other: _______________________

7 RF

9 DK__
